# Primary esophageal mucosa-associated lymphoid tissue lymphoma: A case report and review of literature

**DOI:** 10.1097/MD.0000000000006478

**Published:** 2017-03-31

**Authors:** Qiang Ma, Chun Zhang, San’gao Fang, Peng Zhong, Xiangfeng Zhu, Li Lin, Hualiang Xiao

**Affiliations:** a Department of Pathology, Daping Hospital and Research Institute of Surgery, the Third Military Medical University; b Department of Pathology, Chongqing Corps Hospital of Chinese People's Armed Polices, Chongqing, P.R. China.

**Keywords:** centrocyte-like cell, esophageal, follicular colonizations, *H pylori*, lymphoepithelial lesions, MALT lymphoma

## Abstract

**Rationale:**

Mucosa-associated lymphoid tissue (MALT) lymphoma is a low grade malignant B cell lymphoma which occurs mainly in the organs having mucosal layer. Though gastrointestinal tract is the most commonly involved extranodal site, primary esophageal MALT lymphoma is very rare with less than 20 cases reported in literature.

**Patient concerns:**

A 75-year-old man was referred to our hospital for evaluation of dysphagia. Endoscopy revealed a submucosal tumor located in the middle and lower third of esophagus. CT chest and endoscopic ultrasound revealed a 15.5 × 5.9 × 4.0 cm well circumscribed submucosa esophageal tumor. Test for serum antibody against *H. pylori* was negative. Due to the large tumor size, patient underwent surgical resection. Histological examination showed a submucosal tumor consisting of multiple nodules of varying sizes with intact covering squamous epithelium. The nodules were mainly composed of diffusely and monoclonal proliferating centrocyte-like or monocyte-like cells. Follicular colonizations were observed without lymphoepithelial lesions. The tumor cells were diffusely positive for CD20, PAX-5, Bcl-2 and follicular dendritic cells were positive for CD21, CD23. Monoclonal gene rearrangement was positive for immunoglobulin heavy chain gene, Kappa light chain gene and Lambda light chain gene.

**Diagnoses:**

Based on these findings, final diagnosis of esophageal MALT lymphoma was made.

**Outcomes:**

At 8 month follow up, no recurrence or metastases was detected.

**Lessons:**

Esophageal MALT lymphoma is a rare disease with definitive diagnosis possible only after histopathological examination. It carries good prognosis due to low malignant potential.

## 1 Introduction

The gastrointestinal tract is the most common extranodal site for lymphoma.^[1]^ The 1st case of mucosa-associated lymphoid tissue (MALT) lymphoma at an extranodal site was reported in 1983.^[2]^ Stomach is the most common organ to be affected by MALT lymphoma.^[3]^ Apart from stomach, this lymphoma can affect virtually every other organ, including lungs,^[4]^ intestine,^[5]^ thyroid,^[6]^ thymus,^[7]^ salivary,^[8]^ and skin.^[9]^ Primary esophageal lymphoma is extremely rare, accounting for less than 1% of gastrointestinal cases.^[10]^ Furthermore, based on our literature review, there are less than 20 reported cases of esophageal MALT lymphoma, most of which have been reported from Japan. Histopathology examination of the endoscopic biopsy and/or resected specimen remains the mainstay of diagnosis. Here, we describe a case of esophageal MALT lymphoma with special attention on the tumor morphology, immune phenotyping, and gene rearragements analysis.

## 2 Case report

**Figure 1:**
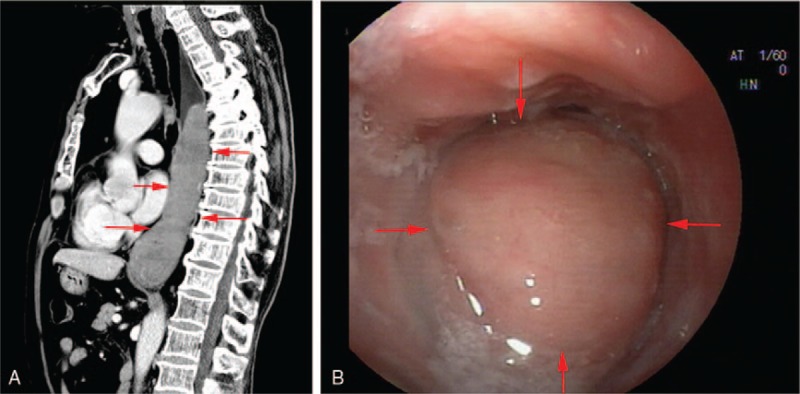
Representative images of computed tomography (CT) scan and endoscopic examination. (A) CT scan showed a huge well-circumscribed and homogeneous ellipsoidal mesenchymal neoplasm in the middle and lower esophageal section. (B**)** Endoscopic examination revealed an oval shaped lesion protruding in to the esophageal lumen with smooth-surface starting at 20 cm from the incisor teeth and extending up to cardia.

A 75-year-old man was referred to our hospital for the evaluation of dysphagia for 9 months. He had associated symptoms of nausea and vomiting but denied hematemesis, hematochezia, lethargy, and dyspnea. He was an active smoker and consumed alcohol for 30 years. Physical examination revealed no peripheral lymphadenopathy, thyromegaly, ascites, or mass in the abdomen. Laboratory data were normal: HBs-Ag negative, HIV negative, and syphilis negative. Test for serum antibody against *Helicobacter pylori* was negative. Chest computed tomography (CT) was notable for a huge well-circumscribed and homogeneous cylindrical mesenchymal neoplasm measuring 15.5×5.9×4.0 cm in lower and middle esophagus with mild contrast enhancement (Fig. 1A). CT revealed no abnormalities in the lungs, heart, ribs, or mediastinum. Endoscopic examination showed a submucosal lesion in the esophagus starting at 20 cm from the incisor teeth extending up to the cardia (Fig. 1B). Endoscopic ultrasonography (EUS) revealed hypoechoic lesion with a clear boundary located in the 4th layer. The mass appeared as a benign tumor and was preoperatively diagnosed as esophageal leiomyoma based on chest CT, endoscopic examination, and EUS findings.

Endoscopic mucosal resection or endoscopic submucosal resection was not possible because of large size. Hence, surgical resection was planned and informed written consent was taken. The patient underwent thoracoscopic-assisted resection of the mass with gastroesophageal anastomosis, thoracic duct ligation, and jejunostomy. Postoperative course was uneventful.

**Figure 2:**
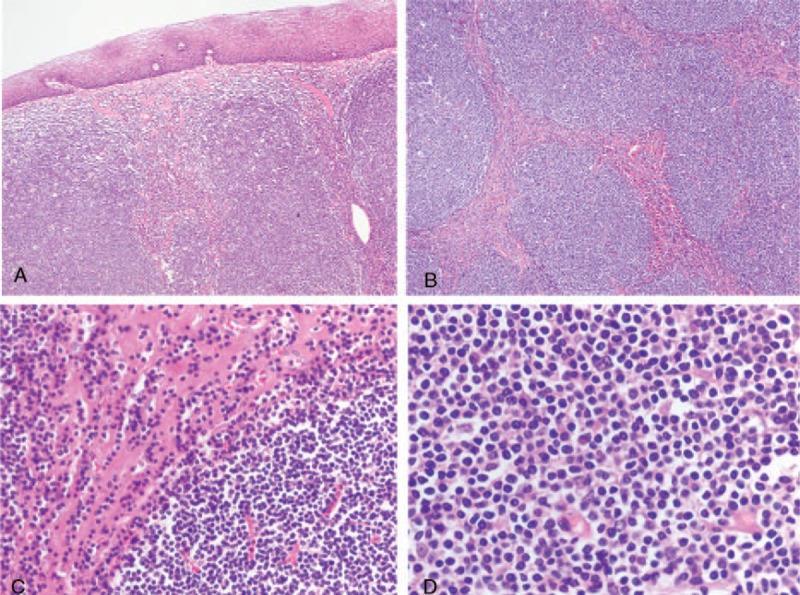
Histologic characteristics of tumor on hematoxylin and eosin (H&E) staining. (A) Tumor was covered with intact squamous epithelium without lymphoepithelial lesions (×40). (B) Tumor was separated into many nodules of varying sizes by collagen fibers (×40). (C) Collagen fibers were infiltrated by cytoplasmic-like cells (×200). (D) The nodules were mainly composed of small to mid-sized centrocyte-like or monocyte-like cells arranged in diffuse pattern (×400).

Grossly, a spindle shaped lump measuring 14 × 3.5 × 2.5 cm, was observed in the resected esophagus, which grew into the esophageal lumen and blocked most of lumen. This lump located in the submucosa was covered with intact mucosa, without erosion, ulcer, and hemorrhage. Its cut surface was homogenously white to grayish-white in color. Histological examination using hematoxylin and eosin staining revealed that tumor was covered with intact squamous epithelium, arising from the submucosal layer and expanding in to the muscular layer. The tumor was composed of many nodules of varying sizes separated by collagen fibers. Numerous cytoplasm-rich cells were observed in the collagenous septations with invasive growth pattern (Fig. 2A–C). The nodules were mainly composed of small to mid-sized centrocyte-like or monocyte-like cells arranged in diffuse pattern. These atypical lymphocytes possessed clear boundary, pale cytoplasm, irregular nucleus, and occasional nucleolus (Fig. 2D). Mitosis was rare. No lymphoepithelial lesion was recognized in the lesion.

**Figure 3:**
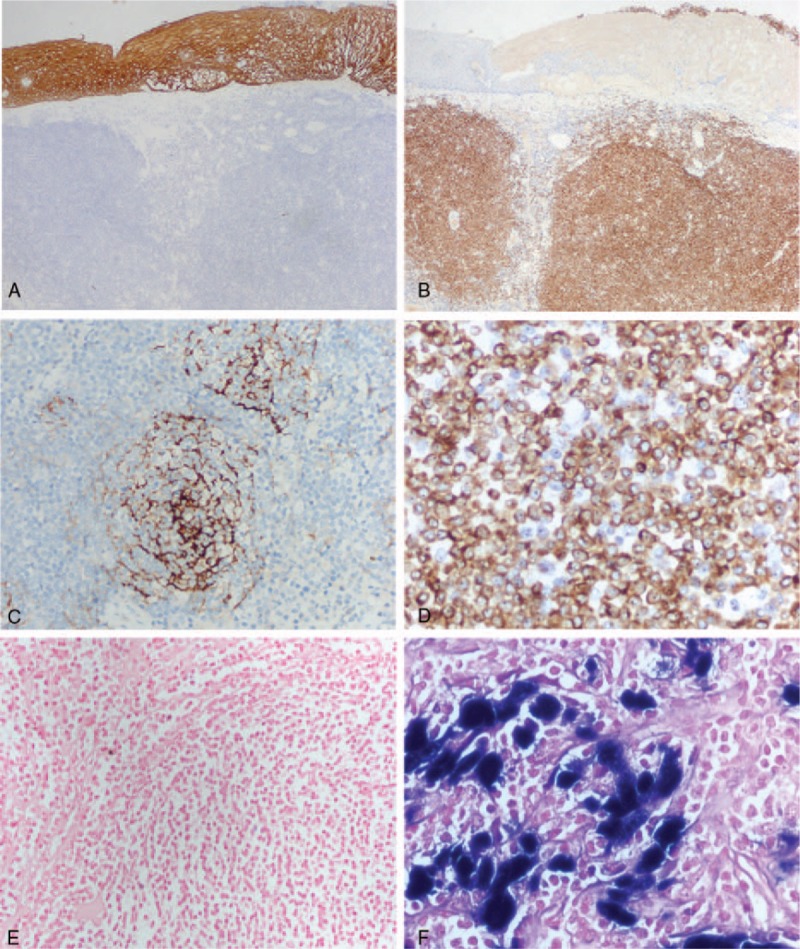
Representative images of immunohistochemical and EBER staining. (A) CK staining of the esophageal epithelium (×40). (B) PAX 5 positivity in tumor cells (×40). (C) CD23 positive follicular dendritic cells (×200). (D) Bcl-2 positive tumor cells (×400). (E) EBER negative tumor cells (×200). (F) EBER positive control in nasopharyngeal carcinoma (×400). Bcl = B-cell lymphoma, CD = cluster of differentiation, CK = cytokeratin, EBER = EB virus-encoded small RNA, PAX 5 = paired box 5.

On immunohistochemical staining, epithelium was diffusely positive for cytokeratin (Fig. 3A), and the tumor cells were diffusely positive for cluster of differentiation (CD)20, paired box 5 (Fig. 3B), and B-cell lymphoma (Bcl)-2 (Fig. 3D). Small deposits of tumor cells, which were distributed mainly in the collagen fibers, were positive for multiple myeloma oncogene 1, CD138, and CD43. Several tumor cells were also positive for CD30. Small lymphocytes were positive for CD3 and CD5. Follicular dendritic cells were positive for CD21 and CD23 (Fig. 3C). All cells were negative for CyclinD1, CD10, Bcl-6, and B lymphocyte specific activation of OCT binding protein 1. These follicular dendritic cells were arranged in nodules in which the tumor cells were relatively evenly distributed. This pattern was suggestive of follicular colonizations seen in MALT lymphoma.

**Figure 4:**
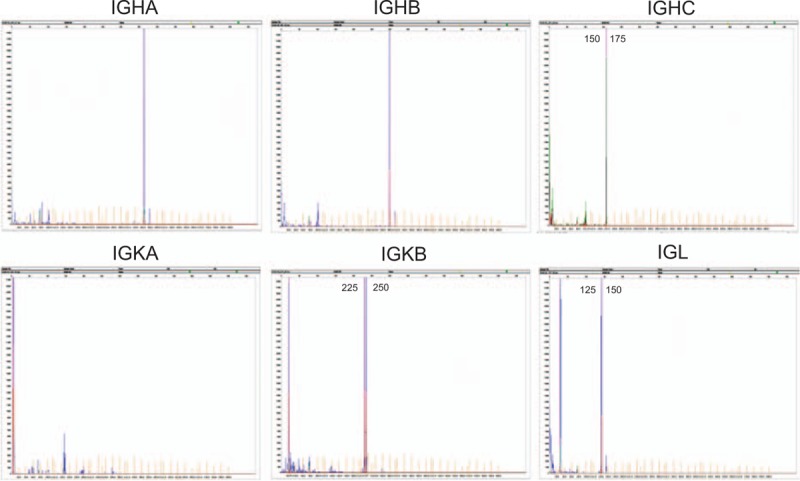
Gene rearrangements and clonality analysis. One band within 150 to 175 base pairs (bp) in heavy chain gene, 2 discrete bands within 225 to 250 bp in kappa light chain gene, and 1 band within 125 to 150 bp in lambda light chain gene.

In this case, gene rearragements and clonality analysis of immunoglobulin heavy chain gene, kappa light chain gene, and lambda light chain gene was performed using the IdentiCloneTM IGH/IGK/IGL Gene Clonality Assay (InVivoScribe Technologies, CA). On monoclonal gene rearrangement, 1 band appeared within 150 to 175 base pairs (bp) in heavy chain gene, 2 discrete bands within 225 to 250 bp in kappa light chain gene, and 1 band within 125 to 150 bp in lambda light chain gene (Fig. 4). In addition, no Epstein–Barr virus was observed in this lymphoma on in situ hybridization using the EB virus-encoded small RNA (Fig. 3E, F).

Based on the clinical data, pathological, immunohistochemical, and gene rearragements analysis, final diagnosis of primary esophageal MALT lymphoma was made. After the surgical resection, no additional therapy in the form of chemotherapy or radiotherapy was administered. Over the 8 months of follow-up, no evidence of recurrence or metastases was found on CT and the patient has been asymptomatic.

Ethical approval for this study was obtained from Medical Ethics Committee of the Third Affiliated Hospital, the Third Military Medical University. Written informed consent was obtained from the patient for the publication of this case report and the accompanying images.

## 3 Discussion

MALT lymphoma, a low grade malignant B cell lymphoma, is mainly seen at the mucosal sites which possesses marginal zone B cells. It has the possibility to transform into highly malignant diffuse large B lymphoma. According to the previous reports, stomach is the most common site of MALT lymphomas. A total of 80% to 90% of gastric MALT lymphomas were found to be associated with *H pylori* infection.^[11]^ Moreover, t(11;18)(q21;q21)-negative gastric MALT lymphomas have shown complete response to *H pylori* eradication.^[12]^ However, to date there is no evidence to suggest association between esophageal MALT lymphoma and *H pylori* infection. As per the 17 reported cases of esophageal MALToma till date (Table 1), 6 cases were positive while 11 cases were negative for *H pylori* infection. No *H pylori* infection was found in our case. On the other hand, it is argued that some other factors may lead to the development of this neoplasia. Structured lymphatic tissue or lymphatic follicles are lacking in normal esophageal mucosa. But due to persistent chronic inflammation, lymphatic follicles may appear and accumulate in the esophageal mucosa for MALT development. Moreover, autoimmune disorders, such as Sjogren disease,^[13]^ Hashimoto disease,^[14]^ are also believed to cause MALT lymphoma in previous reports. However, its pathogenesis is far from being clear due to the rarity of this disease.

Based on our literature review of 17 cases, patients with esophageal MALT lymphoma were aged between 49 and 83 years (mean, 63 years), with male:female ratio of 12:5. Dysphagia and sensation of foreign body were the most common symptoms. Some had non-specific symptoms and the esophageal lesion was detected during a routine medical check-up.^[15]^ Several approaches, such as endoscopic examination, endosonography of the upper gastrointestinal tract, and chest CT scan, were used to examine the esophageal lesion in clinical practice. However, it was difficultly to confirm the diagnosis of esophageal MALT lymphoma through these methods. In our case the tumor was misdiagnosed as esophageal leiomyoma on chest CT and EUS. Therefore, the final diagnosis depends upon detailed histological examination.

MALT lymphoma in esophagus is morphologically similar to that of other sites. Esophageal MALT lymphoma mainly presents as diffuse growth in the submucosal layer, with or without infiltration into the muscular layer. Proliferating tumor cells show moderate atypia, clear cytoplasm, and irregular nuclear membrane. They are morphologically classified in to 3 types: centrocyte-like cells, monocyte-like cells, and small lymphocyte-like cells. These 3 types can exist in isolation or can be found together in different proportions. In some cases, tumor cells invade into reactive hyperplastic follicular germinal centers resulting in the replacement of the germinal centers in to a multinodular structure. In follicular colonization, the tumor cells (CD20+, Bcl-2+, CD10−, and Bcl-6−) colonize the reactive follicles composed of dendritic cells (CD21+, CD23+) with distorting the original follicular structure (Fig. 3). When proliferating tumor cells infiltrate and destroy the epithelial structure, they form “lymphoepithelial lesions,” which are commonly observed in gastric MALT lymphoma, but were found in only 3 out of 17 reviewed cases of esophageal MALToma (Table 1). It is distinguished from follicular lymphoma by negativity for CD10 and Bcl-6, from mantle cell lymphoma by negativity for CD5 and Cyclin D1, and from nodular sclerosing Hodgkin lymphoma by lack of CD30-positive lacunar cells (Reed–Sternberg cells).

The clinical features and biological behavior in MALT lymphoma has similarity with chronic inflammation. Particularly, the morphological characteristics of chronic inflammation and early MALT lymphoma in some cases are difficult to differentiate on histopathology. Fortunately, with the improvement in molecular techniques, gene rearrangement and clonality analysis have been used to differentiate them based on the hypothesis that immunoglobulin gene rearrangements occur in B cell tumorigenesis, and not in chronic inflammation.^[16]^ In our case, monoclonal gene rearrangement analysis revealed positivity for immunoglobulin heavy chain gene, kappa light chain gene, and lambda light chain gene, confirming it to be a tumor, not inflammation.

Recent studies have demonstrated several chromosomal abnormalities in MALT lymphoma. Among them, t(11; 18)(q21;q21) was the first to be discovered followed by t(1; 14)(p22; q32), t(14; 18)(q32;q21), t(3; 14)(p14.1;q32), aneuploidy (Trisomy 3, 18), and TNFAIP3 abnormality.^[12,17–22]^ Importantly, the diagnosis, treatment, and prognosis of MALT lymphoma with different chromosomal abnormalities are different. Therefore, the molecular classification of MALT lymphoma will be a general trend in lymphoma development.

*H pylori* eradication therapy has been confirmed to cause regression of t(11;18)(q21;q21)-negative gastric MALT lymphoma, but not reported in esophageal MALT lymphoma. Hence, whether or not use of *H pylori* eradication therapy on the treatment of esophageal MALToma needed a further research. In 17 reviewed cases (Table 1), masses with 3 cm or above in maximum diameter received surgical resection and that with 3 cm below in maximum diameter received endoscopic mucosal resection or endoscopic submucosal resection local excision. Besides, radiotherapy or chemotherapy was the common treatment strategy. However, the best treatment strategy is not known due to its rare incidence and slow disease progression. However, irrespective of the treatment modality, patients have good prognosis as MALT lymphoma in itself is a low grade malignant tumor.

**Table 1:**
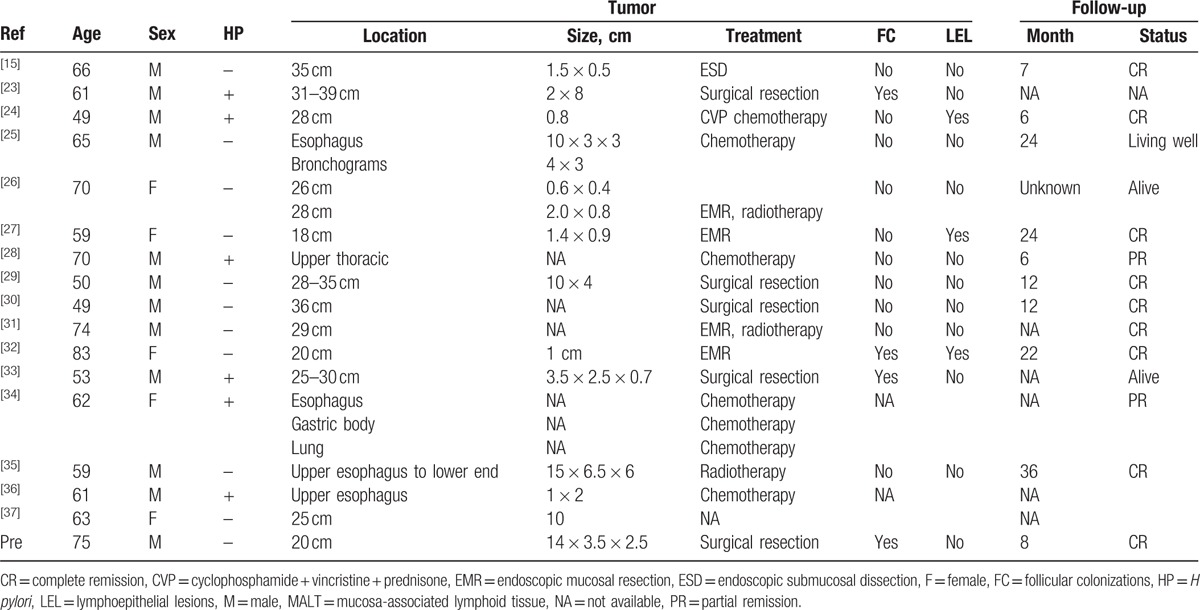
Cases of esophageal MALT lymphoma reported in previous literatures and the current case.

## 4 Conclusion

Esophageal MALT lymphoma is a rare disease presenting as submucosal tumor. Definitive diagnosis is possible only with detailed histopathological examination including immune phenotyping and gene rearrangement analysis. The best treatment strategy is not known as chemotherapy, radiotherapy, endoscopic, and surgical resection has been used with similar success. The overall prognosis of MALT lymphoma in esophagus is good due to low malignant potential.

## Acknowledgements

The authors thank the patient for agreeing with our report and for providing a detailed medical history. The authors also thank Medjaden Bioscience Limited for manuscript editing and proofread.
